# Associations of Head and Neck Cancer with Hepatitis B Virus and Hepatitis C Virus Infection

**DOI:** 10.3390/cancers15184510

**Published:** 2023-09-11

**Authors:** Shih-Han Hung, Tzong-Hann Yang, Yen-Fu Cheng, Chin-Shyan Chen, Herng-Ching Lin

**Affiliations:** 1 Department of Otolaryngology, School of Medicine, Taipei Medical University, Taipei 110, Taiwan; seedturtle@gmail.com; 2 Department of Otolaryngology, Wan Fang Hospital, Taipei 110, Taiwan; 3 International Ph.D. Program in Medicine, College of Medicine, Taipei Medical University, Taipei 110, Taiwan; 4 Department of Otorhinolaryngology, Taipei City Hospital, Taipei 110, Taiwan; tzonghannyang@gmail.com; 5 Department of Speech, Language and Audiology, National Taipei University of Nursing and Health, Taipei 112, Taiwan; 6 Department of Otolaryngology-Head and Neck Surgery, School of Medicine, National Yang Ming Chiao Tung University, Taipei 112, Taiwan; entist@gmail.com; 7 Center of General Education, University of Taipei, Taipei 112, Taiwan; 8 Research Center of Sleep Medicine, College of Medicine, Taipei Medical University, Taipei 110, Taiwan; stan@mail.ntpu.edu.tw; 9 Department of Medical Research, Taipei Veterans General Hospital, Taipei 112, Taiwan; 10 Department of Otolaryngology-Head and Neck Surgery, Taipei Veterans General Hospital, Taipei 112, Taiwan; 11 Department of Economics, National Taipei University, New Taipei City 112, Taiwan; 12 School of Health Care Administration, College of Management, Taipei Medical University, Taipei 110, Taiwan; 13 Research Center of Sleep Medicine, Taipei Medical University Hospital, Taipei 110, Taiwan

**Keywords:** head and neck cancer, epidemiology, hepatitis B virus, hepatitis C virus

## Abstract

**Simple Summary:**

This case-control study investigates the associations between head and neck cancer (HNC), hepatitis B virus (HBV), and hepatitis C virus (HCV) infection. We included 5603 patients who had received a diagnosis of HNC as cases and 16,809 propensity score matching controls. Results suggest a significant difference in the prevalence of HBV infection and HCV infection between cases and controls. Our study provides evidence that suggests a potential association between HBV and HCV infections and the risk of HNC.

**Abstract:**

This case-control study investigates the associations between head and neck cancer (HNC), hepatitis B virus (HBV), and hepatitis C virus (HCV) infection. We included 5603 patients who had received a diagnosis of HNC as cases and 16,809 propensity score matching controls. We employed multivariate logistic regression models to evaluate the association of HNC with HBV and HCV infection after taking sociodemographic characteristics and diabetes, hypertension, hyperlipidemia, HPV infection, tobacco use disorder, and alcohol abuse/alcohol dependence syndrome into considerations. Results show that 7.9% of the total sample had been previously diagnosed with HBV infection, with 9.0% prevalence among cases and 7.6% among controls (*p* < 0.001). The chi-squared test suggests a significant difference in the prevalence of HCV infection between cases and controls (3.3% vs. 2.7%, *p* = 0.019). The covariate-adjusted odds ratio (OR) of HBV infection in patients with HNC relative to controls was 1.219 (95% CI = 1.093~1.359). Additionally, the adjusted OR of HCV infection in patients with HNC was 1.221 (95% CI = 1.023~1.457) compared to controls. Furthermore, patients with oropharyngeal cancer were more likely to have HCV infection than controls (adjusted OR = 2.142, 95% CI = 1.171~3.918). Our study provides evidence that suggests a potential association between HBV and HCV infections and the risk of HNC.

## 1. Introduction

Head and neck cancer (HNC) comprises diverse malignancies affecting the upper aerodigestive tract, including the oral cavity, pharynx, and larynx [1]. Globally, HNC represents the sixth most common cancer, with an estimated 890,000 new cases and 450,000 deaths annually [2]. The primary risk factors for HNC include tobacco and alcohol use and infection with high-risk strains of human papillomavirus (HPV) [3]. 

The role of viruses in the oncogenesis of head and neck cancer remains largely unexplored. There are eight recognized human oncoviruses, including the hepatitis B virus (HBV) and the hepatitis C virus (HCV), which have strong evidence for their causality of different cancers. It is estimated that approximately 10.2% of cancers worldwide are virally driven, equating to over 1,968,600 cases annually [4]. In recent years, there has been increasing interest in understanding the potential role of viral hepatitis, specifically HBV and HCV, in the pathogenesis of HNC. Besides liver diseases, these viruses also have well-documented extrahepatic manifestations, including lymphoproliferative disorders and non-Hodgkin lymphoma [5]. Several epidemiological studies have suggested a possible link between HCV infection and non-liver malignancies, including HNC [6]. Emerging evidence also points to a potential role of HCV in HNC, although the data are less robust [7,8]. Given the significant global burden of HBV and HCV infections and the severe outcomes associated with HNC, it is critical to further elucidate these potential associations.

This study investigates the associations between HNC, HBV, and HCV infection. We hypothesize that there is a significant association between these viral infections and HNC, and that they may contribute to the risk of HNC. A better understanding of these associations could have significant implications for the prevention, early detection, and treatment of HNC, potentially improving patient prognosis and survival.

## 2. Materials and Methods

### 2.1. Database

We utilized data from Taiwan’s Longitudinal Health Insurance Database 2010 (LHID2010), a retrospective observational study. Since 1995, Taiwan has operated a single-payer mandatory social healthcare insurance system. The Taiwan National Health Insurance (NHI) program offers comprehensive medical coverage with low co-payment for all Taiwanese citizens. The LHID2010 database contains beneficiary registration and medical claim files for a randomly selected sample of 2,000,000 NHI beneficiaries. The database has been extensively used by a variety of researchers and scholars for epidemiological investigations of diseases and treatments.

This study obtained approval from the Institutional Review Board of Taipei Medical University (TMU-JIRB N202208042) and is compliant with the Declaration of Helsinki. As this study utilized de-identified administrative data, patient informed consent was waived.

### 2.2. Identification of Study Patients

For this case-control study, the initial patient group comprised 5603 individuals aged 20 years or older who received a first-time diagnosis of HNC. The diagnoses included various types of cancers, identified using their respective International Classification of Diseases (ICD) codes, including cancers of the oral cavity (ICD-9-CM codes 140, 141 (except 141.0 and 141.6), 143, 144, 145.2, 145.3, 145.5, and 145 (except 145.2; 145.3; and 145.5), ICD-10-CM codes C00 and C02 (except C02.4; C03; C04; C05; and C06), oropharynx (ICD-9-CM 141.0, 141.6, and 146, ICD-10-CM C01, C02.4, C09, and C10), larynx (ICD-9-CM 161 and ICD-10-CM C32), hypopharynx (ICD-9-CM 148, ICD-10-CM C12, and C13), nasopharynx (ICD-9-CM 147 and ICD-10-CM C11), sinonasal (ICD-9-CM 160 (except 160.1), ICD-10-CM C31 and C30.0), salivary gland (ICD-9-CM 142 and ICD-10-CM codes C07 and C08) and thyroid (ICD-9-CM 193 and ICD-10-CM code C73), during the period from 1 January 2015 to 31 December 2019. The date of their first-time HNC diagnosis was established as the index date.

To examine the association of HNC with HBV and HCV infection, we employed the propensity score matching method to select controls from the remaining beneficiaries aged 20 years and above from the Registry of Beneficiaries of LHID2010. All enrollees who had a history of HNC in a medical claim were excluded. Propensity scores were calculated for all 5603 selected patients with HNC and remaining beneficiaries using the logistic regression model with adjustment for age, sex, monthly income (TWD) 0~15,840, TWD 15,841~25,000, ≥TWD 25,001; USD 1 ≈ TWD 28 in 2021), geographic location (northern, central, southern, and eastern), and urbanization level of the patient’s residence (5 levels, 1 = most urbanized, 5 = least urbanized); and diabetes, hypertension, hyperlipidemia, and human papillomavirus (HPV) infection. Finally, each sampled patient with HNC was matched for three controls without HNC using the nearest neighbor random matching algorithm with caliper adjustment, using a priori value for the calipers of +/−0.01. While for the cases, we assigned the year of the index date as the year in which the cases received their first HNC diagnosis. For controls, the year of the index date was simply a matched year in which controls had an ambulatory care visit. As a result, this study’s sample was composed of 5603 patients with HNC and 16,809 controls without HNC.

### 2.3. Measures of Outcomes

We identified cases with HBV infection based on ICD-9-CM codes 070.20, 070.21, 070.22, 070.23, 070.30, 070.31, 070.32, 070.33, and V02.61, or ICD-10-CM codes B16, B16.0, B16.1, B16.2, B16.9, B17.0, B18.0, B18.1, B19.2, and Z22.51. We identified cases with HCV infection based on ICD-9-CM codes 070.41, 070.44, 070.51, 070.54, 070.70, 070.71, and V02.62, or ICD-10-CM codes B15, B17.1, and B18.2. Inclusion in this study required at least one diagnosis of HBV and HCV infection prior to the index date.

### 2.4. Statistical Analysis

The SAS System (SAS System for Windows, vers. 9.4, SAS Institute, Cary, NC, USA) was used for all statistical analyses. We employed chi-square tests and *t*-tests to investigate differences in baseline characteristics between HNC patients and controls. Moreover, multivariate logistic regression models were utilized to quantitatively evaluate the association of HNC with HBV and HCV infections after adjusting for the age, sex, monthly income, geographic location, and urbanization level of the patient’s residence, diabetes, hypertension, hyperlipidemia, HPV infection, tobacco use disorder, and alcohol abuse/alcohol dependence syndrome. The odds ratio (OR) and the 95% confidence intervals (CIs) were used to quantify the difference in odds of HBV and HCV between HNC patients and controls. A *p*-value < 0.05, from two-sided tests, was deemed statistically significant.

## 3. Results

The sociodemographic characteristics and medical comorbidities among the sample patients are presented in Table 1. The mean age was found to be statistically identical for cases and controls, with an average of 56.3 ± 13.4 years and 56.4 ± 13.4 years, respectively, as the *p*-value equaled 0.730. There was no statistically significant difference between cases and controls in terms of sex (*p* > 0.999), monthly income (*p* = 0.968), geographic location (*p* = 0.245), and the level of urbanization (*p* > 0.999) in their sociodemographic characteristics. Similarly, in terms of medical comorbidities, no significant differences were observed between cases and controls in the occurrence of diabetes (26.1% in both groups, *p* > 0.999), hyperlipidemia (34.1% in both groups, *p* > 0.999), hypertension (41.5% in both groups, *p* > 0.999), and HPV infections (2.5% in both groups, *p* > 0.999). There were statistically significant differences in the prevalence of tobacco use disorder (*p* < 0.001) and alcohol abuse/alcohol dependence syndrome (*p* < 0.001) between cases and controls.

Table 2 highlights the prevalence of HBV infection among cases and controls, showing that 7.9% (equivalent to 5603) of the total sample had been previously diagnosed with the infection, with a 9.0% prevalence among cases and 7.6% among controls (*p* < 0.001). Moreover, there were significant differences in the prevalence of HBV infection between patients with hypopharynx cancer (11.1% vs. 7.6%, *p* = 0.019), nasopharynx cancer (11.0% vs. 7.6%, *p* < 0.001), and thyroid cancer (9.9% vs. 7.6%, *p* = 0.001) compared to controls.

The prevalence of HCV infection among cases and controls is represented in Table 3. The chi-squared test suggests a significant difference in the prevalence of HCV infection between cases and controls (3.3% vs. 2.7%, *p* = 0.019). Significant differences were also observed in the prevalence of HCV infection between patients with oropharynx cancer (4.8% vs. 2.7%, *p* = 0.007) and thyroid cancer (3.4% vs. 2.7%, *p* = 0.010) when compared with controls.

**Figure 1:**
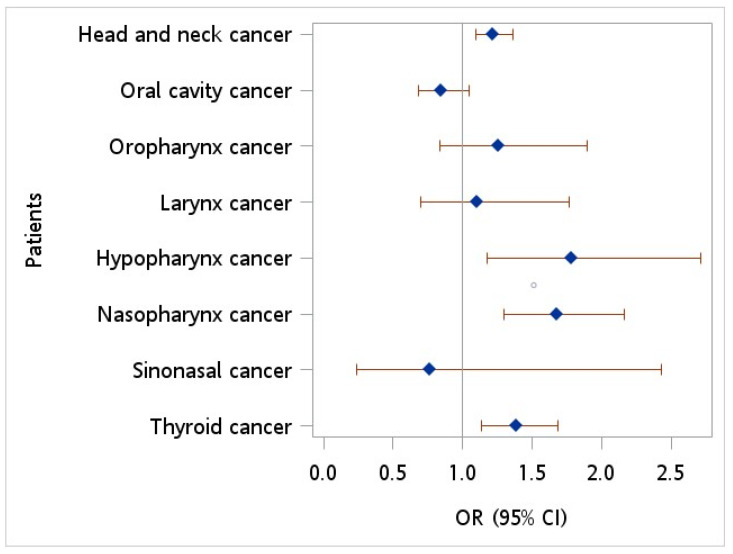
Odds ratio of hepatitis B virus among patients with head neck cancer vs. controls after adjusting for age, sex, monthly income, geographic location, urbanization level, hypertension, diabetes, hyperlipidemia, HPV infection, tobacco use disorder, and alcohol abuse.

Figure 1 provides the covariate-adjusted OR for HBV infection for HNC patients relative to controls. After adjustment for age, sex, monthly income, geographic location, urbanization level, hypertension, diabetes, hyperlipidemia, HPV infection, tobacco use disorder, and alcohol abuse/alcohol dependence syndrome, an OR of 1.219 (95% CI = 1.093~1.359, *p* < 0.001) was observed. Furthermore, multiple logistic regression demonstrated an increased OR in HBV infection of 1.785 (95% CI = 1.174~2.712, *p* = 0.007), 1.676 (95% CI = 1.301~2.160, *p* < 0.001), and 1.383 (95% CI = 1.133~1.688, *p* = 0.001) in patients with hypopharynx cancer, nasopharynx cancer, and thyroid cancer, respectively, relative to controls.

**Figure 2:**
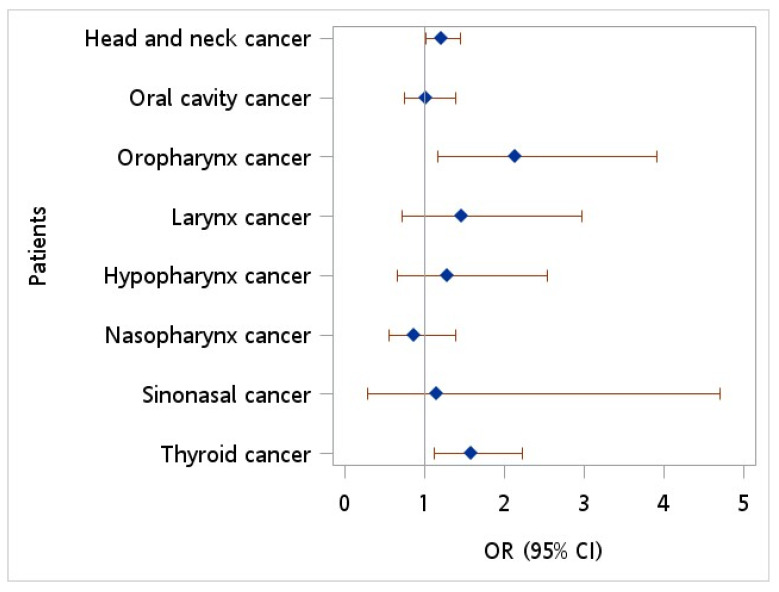
Odds ratio of hepatitis C virus among patients with head neck cancer vs. controls after adjusting for age, sex, monthly income, geographic location, urbanization level, hypertension, diabetes, hyperlipidemia, HPV infection, tobacco use disorder, and alcohol abuse.

Figure 2 depicts the ORs of HCV infection for HNC patients versus controls. An OR of 1.221 (95% CI = 1.023~1.457, *p* = 0.027) was observed in patients with HNC when compared to controls after adjusting for monthly income, geographic location, urbanization level, diabetes, hypertension, hyperlipidemia, HPV infection, tobacco use disorder, and alcohol abuse/alcohol dependence syndrome. Additionally, patients diagnosed with oropharynx cancer exhibited a greater likelihood of HCV infection than controls, with an adjusted OR of 2.142 (95% CI = 1.171~3.918, *p* = 0.013).

## 4. Discussion

In this study, we investigated the association between HBV and HCV infections and the risk of HNC. Our study found a significant association between HCV infection and HNC, particularly in patients with oral cavities, oropharynx, larynx, and thyroid cancers. Specifically, we found that patients with HNC had a higher prevalence of HBV and HCV infections than controls. The odds ratio (OR) of HBV infection in patients with HNC was 1.219 (95% CI = 1.093~1.359), and the OR of HCV infection was 1.221 (95% CI = 1.023~1.457) after adjusting for confounding factors. The findings align with the growing body of research that suggests a potential role of viral hepatitis in the pathogenesis of HNC.

Understanding the role of hepatitis viruses in the pathogenesis of HNC is crucial as it provides insights into the etiology of HNC and also highlights the potential for viral hepatitis as a risk factor for HNC. Previous studies have suggested a potential link between HCV infection and non-liver malignancies, including HNC [9]. Nyberg et al. reported that cancer burden (all sites) was significantly higher in HCV than in non-HCV patients, and HCV patients had a high rate of liver cancer. When liver cancer was excluded, cancer rates remained significantly increased in HCV [10]. Emerging evidence also points to a potential role of HBV in HNC. A study by Chen et al. found that HBV infection was associated with an increased risk of HNC, particularly oropharyngeal and hypopharyngeal cancers [11]. Another study by Mahale et al. reported that HCV is associated with non-oropharyngeal (except nasopharyngeal) and HPV-positive oropharyngeal HNCs [7]. A study investigating HBV and HCV in head and neck squamous cell carcinoma (HNSCC) patients demonstrated a positive association between hepatitis B and hepatitis C chronic infection and HNSCC [8]. The authors suggested that an awareness of the possibility of the increased risk of HNSCC may lead to earlier diagnosis and better outcomes in patients with hepatitis B and C. 

Several possible mechanisms might help to explain this possible association. 

The HCV infection may contribute to the pathogenesis of HNC through oxidative stress. Oxidative stress is produced by inflammatory progressions that occur in hepatitis via immunological mechanisms. In HCV infection, reactive oxygen species (ROS) are produced with NADPH oxidase and xanthine oxidase in neutrophils and macrophages [12]. A study by Machida et al. found that HCV proteins, particularly the Core protein, cause oxidative stress, which can lead to the development of HNC [13,14]. The mechanisms by which HCV can lead to HNC involve the induction of oxidative stress and steatosis, the activation of specific cellular genes with carcinogenic effects, and the upregulation of ROS production by HCV’s Core protein. These mechanisms could potentially contribute to developing other types of cancer, including head and neck cancer [15].

Little has been addressed regarding the possible mechanisms linking HBV and head and neck cancer carcinogenesis. A study by Yan et al. provides insights into the mechanism with which HBV can induce cancer, including head and neck cancer [16]. This study suggests that the hepatitis B virus X protein (HBx) plays a significant role in the progression of hepatocellular carcinoma (HCC) and highlights the role of a protein called PTPN13, which is known to regulate FAS-induced apoptosis and NGFR-mediated proapoptotic signaling negatively. PTPN13 is involved in cancers such as colorectal, breast, lymphomas, and head and neck squamous cell carcinoma. Their study found that HBx downregulates PTPN13 expression, which negatively affects cell proliferation and tumorigenesis by interfering with the function of IGF2BP1. While this study focuses on hepatocellular carcinoma, the mechanisms described could potentially apply to other types of cancer, including head and neck cancer, as PTPN13 is also involved in head and neck squamous cell carcinoma [17,18,19].

The findings of our study have several clinical implications. The significant association between HBV and HCV infections and the occurrence of HNC suggests that these viral infections could contribute to the development of HNC. If these associations are confirmed in further studies, individuals with HBV or HCV infection may benefit from screening for HNC. Also, it could lead to the implementation of routine screening for HBV and HCV in patients at risk of HNC. This would allow for early detection and treatment of these viral infections, potentially reducing the incidence of HNC. 

Furthermore, this study’s findings could also open up new avenues for therapeutic intervention. Antiviral therapies, which are currently used to treat HBV and HCV infections, could potentially be incorporated into the treatment regimen for HNC, providing a novel approach to managing this disease. 

A study investigating the effects of antiviral treatment in breast cancer cells provides insights into the potential benefits of antiviral treatment for cancer patients [20]. This study found that the antiviral drug acyclovir (ACV) had a suppressive effect on MCF7 breast cancer cells. It also induced downregulation of ALDH activity, suggesting a decrease in the tumorigenic potential of the treated cancer cells. ALDH is an enzyme often upregulated in cancer cells and associated with poor clinical outcomes.

Furthermore, the treatment with ACV led to an upregulated secretion of E-cadherin, a protein that is essential for cell-to-cell adhesion and whose downregulation often leads to the stimulation of invasion and metastasis. While these findings are promising, this study was performed in vitro and only on a one cell line, so more research is needed to confirm these results and to determine whether similar effects would be seen in head and neck cancer patients. However, these findings suggest that antiviral treatment could be a beneficial addition to managing head and neck cancer [21,22,23,24,25].

It is important to note that while these implications are promising, they are based on observational data, and further research is needed to confirm these associations and explore the underlying mechanisms. Nevertheless, these findings could guide future research on the molecular mechanisms underlying the association between viral hepatitis and HNC and pave the way for innovative strategies for its prevention and treatment. 

Our study has several limitations. First, this study’s design was retrospective and observational in nature, which inherently limits the ability to establish causality between HBV and HCV infections and the development of HNC. The reliance on administrative data and ICD codes for case identification introduces the possibility of misclassification and incomplete information. Additionally, this study’s sample was obtained from Taiwan’s Longitudinal Health Insurance Database, which may not fully represent the global population and might limit the generalizability of the findings. It should be noted that based on this database, whether patients with viral hepatitis were under treatment and the actual viral load before or after the treatment cannot be addressed accurately. Furthermore, this study focused solely on the association between HBV and HCV infections and HNC without exploring the underlying mechanisms or interactions between these viruses and other risk factors. Future studies should consider a prospective design, a larger and more diverse population, comprehensive adjustment for confounders, and the inclusion of other relevant risk factors to strengthen the evidence and provide a more comprehensive understanding of the relationship between viral infections and HNC. 

Moreover, future research should elucidate the molecular mechanisms underlying the association between viral hepatitis and HNC. In particular, studies should investigate how HBV and HCV contribute to oncogenesis in the head and neck. Additionally, prospective cohort studies are needed to confirm our findings and assess the potential benefits of antiviral therapy in reducing the risk of HNC in individuals with viral hepatitis. Despite these limitations, this study provides valuable preliminary evidence and highlights the need for further research.

In light of our findings and the existing literature, we recommend that future guidelines for the management of patients with viral hepatitis should consider the potential risk of HNC. Furthermore, we recommend that future research should focus on elucidating the molecular mechanisms underlying the association between viral hepatitis and HNC, which could lead to the development of targeted preventive and therapeutic strategies. A better understanding of these associations could have significant implications for the prevention, early detection, and treatment of HNC, potentially improving patient prognosis and survival.

## 5. Conclusions

In conclusion, our study provides evidence of a significant association between HBV and HCV infections and the risk of HNC. Clinicians should be aware of this association and consider screening for HNC in individuals with viral hepatitis. Further research is needed to confirm these findings and explore the potential benefits of antiviral therapy in reducing the risk of HNC in individuals with viral hepatitis.

## Figures and Tables

**Table 1:** Demographic characteristics of patients with HNC and propensity score-matched controls.

Variable	Patients with HNC (*n* = 5603)		Propensity Score-Matched Controls (*n* = 16,809)		*p* Value
	Total No.	%	Total No.	%	
Age, mean (SD)	56.3 (13.4)		56.4 (13.4)		0.730
Males	3647	65.1	10,941	65.1	>0.999
Monthly Income					0.968
<TWD 1~15,841	1225	21.9	3657	21.8	
TWD 15,841~25,000	2130	38.0	6377	37.9	
≥TWD 25,001	2248	40.1	6775	40.3	
Geographic region					0.245
Northern	2454	43.8	7368	43.8	
Central	1346	24.0	4049	24.1	
Southern	1632	29.1	4963	29.5	
Eastern	171	3.1	429	2.	
Urbanization level					>0.999
1 (most urbanized)	1434	25.6	4302	25.6	
2	1599	28.5	4795	28.5	
3	984	17.6	2969	17.7	
4	739	13.2	2220	13.2	
5 (least urbanized)	847	15.1	2523	15.0	
Hypertension	2328	41.6	6984	41.6	>0.999
Hyperlipidemia	1893	33.8	5679	33.8	>0.999
Diabetes	1453	25.9	4359	25.9	>0.999
HPV infection	137	2.5	411	2.5	>0.999
Tobacco use disorder	509	9.1	744	4.4	<0.001
Alcohol abuse	131	2.3	152	0.9	<0.001

**Table 2:** Prevalence rates of hepatitis B virus infection among patients with HNC vs. controls.

Variable	Presence of Hepatitis B Virus Infection		Without Hepatitis B Virus Infection		*p* Value
	*n*, %		*n*, %		
Patients with head and neck cancer	503	9.0	5100	91.0	<0.001
Patients with oral cavity cancer	123	6.8	1679	93.2	0.119
Patients with oropharynx cancer	37	8.9	378	91.1	0.342
Patients with larynx cancer	28	9.6	263	90.4	0.679
Patients with hypopharynx cancer	41	11.1	327	88.9	0.019
Patients with nasopharynx cancer	103	11.0	831	89.0	<0.001
Patients with sinonasal cancer	4	4.2	91	95.8	0.684
Patients with salivary gland cancer	-	-	-	-	
Patients with thyroid cancer	156	9.9	1419	90.1	0.001
Controls	1267	7.6	15,542	92.4	

**Table 3:** Prevalence rates of hepatitis C virus infection among patients with HNC vs. controls.

Variable	Presence of Hepatitis C Virus Infection		Without Hepatitis C Virus Infection		*p* Value
	*n*, %		*n*, %		
Patients with head and neck cancer	183	3.3	5420	96.7	0.019
Patients with oral cavity cancer	56	3.1	1746	96.9	0.750
Patients with oropharynx cancer	20	4.8	395	95.2	0.007
Patients with larynx cancer	12	4.1	279	95.9	0.3419
Patients with hypopharynx cancer	13	3.5	355	96.5	0.480
Patients with nasopharynx cancer	24	2.6	910	97.4	0.569
Patients with sinonasal cancer	3	3.2	92	96.8	0.860
Patients with salivary gland cancer	-	-	-	-	-
Patients with thyroid cancer	53	3.4	1522	96.6	0.010
Controls	448	2.7	16,361	97.3	

## Data Availability

Data from the National Health Insurance Research Database, now managed by the Health and Welfare Data Science Center (HWDC), can be obtained by interested researchers through a formal application process addressed to the HWDC, Department of Statistics, Ministry of Health and Welfare, Taiwan (https://dep.mohw.gov.tw/DOS/lp-2506-113.html, accessed on 2 January 2022).
